# Planning for resilience in screening operations using discrete event simulation modeling: example of HPV testing in Peru

**DOI:** 10.1186/s43058-022-00302-5

**Published:** 2022-06-17

**Authors:** Anne F. Rositch, Aditya Singh, Nadia Lahrichi, Valerie A. Paz-Soldan, Anna Kohler-Smith, Patti Gravitt, Erica Gralla

**Affiliations:** 1 Department of Epidemiology, Johns Hopkins Bloomberg School of Public Health, 615 N. Wolfe St, Room E6150, Baltimore, MD 21205 USA; 2 Engineering Management and Systems Engineering, The George Washington University, 800 22nd Street NW, Suite 2680, Washington, D.C., 20052 USA; 3 Mathematics and Industrial Engineering, Polytechnique Montreal, CP6079 Succ. centre-ville, Montreal, H3C 3A7 Canada; 4 Tropical Medicine, Tulane School of Public Health and Tropical Medicine, 1440 Canal Street, Suite 2210, New Orleans, LA 70112 USA; 5 Asociación Benéfica Prisma, Av. Santo Toribio 115. Oficina 701. Urb. El Rosario, San Isidro, Peru; 6 Department of Epidemiology and Public Health, University of Maryland School of Medicine, 655 W. Baltimore Street, Baltimore, MD 21201 USA

**Keywords:** Implementation planning, Discrete event simulation, DES modeling, Cervical cancer elimination, Peru, LMICs, Operations research, Resilience, Disruption

## Abstract

**Background:**

The World Health Organization (WHO) has called for the elimination of cervical cancer. Unfortunately, the implementation of cost-effective prevention and control strategies has faced significant barriers, such as insufficient guidance on best practices for resource and operations planning. Therefore, we demonstrate the value of discrete event simulation (DES) in implementation science research and practice, particularly to support the programmatic and operational planning for sustainable and resilient delivery of healthcare interventions. Our specific example shows how DES models can inform planning for scale-up and resilient operations of a new HPV-based screen and treat program in Iquitos, an Amazonian city of Peru.

**Methods:**

Using data from a time and motion study and cervical cancer screening registry from Iquitos, Peru, we developed a DES model to conduct virtual experimentation with “what-if” scenarios that compare different workflow and processing strategies under resource constraints and disruptions to the screening system.

**Results:**

Our simulations show how much the screening system’s capacity can be increased at current resource levels, how much variability in service times can be tolerated, and the extent of resilience to disruptions such as curtailed resources. The simulations also identify the resources that would be required to scale up for larger target populations or increased resilience to disruptions, illustrating the key tradeoff between resilience and efficiency. Thus, our results demonstrate how DES models can inform specific resourcing decisions but can also highlight important tradeoffs and suggest general “rules” for resource and operational planning.

**Conclusions:**

Multilevel planning and implementation challenges are not unique to sustainable adoption of cervical cancer screening programs but represent common barriers to the successful scale-up of many preventative health interventions worldwide. DES represents a broadly applicable tool to address complex implementation challenges identified at the national, regional, and local levels across settings and health interventions—how to make effective and efficient operational and resourcing decisions to support program adaptation to local constraints and demands so that they are resilient to changing demands and more likely to be maintained with fidelity over time.

Contributions to the literature

- While decision-analytic modeling has proven critical for the adoption of new technologies, our discrete event simulation (DES) models focus on the *next* set of decisions: resources and operations critical to adequate delivery of services.
- As an example, for an HPV-based screening program, our results assess its resilience to variability and disruption and the resources required to scale up capacity.
- These operational aspects of implementation have received limited attention but are critical in moving evidence-based interventions into routine practice.
- Thus, we demonstrate that DES models fill an essential and neglected role in translating new technologies and interventions into practice.

## Introduction

The World Health Organization (WHO) has called for the elimination of cervical cancer through accelerated implementation of cost-effective prevention and control strategies, including screening with the treatment of precancerous lesions [1]. Low- and middle-income countries (LMICs), which bear the largest burden of cervical cancer and currently have either no, or highly ineffective, screening programs, can meet this global call to action only if significant efforts are made to adopt screening programs designed specifically for resource-limited settings [2]. Thus, the WHO guidelines now suggest choosing among several cost-effective screening strategies based on the availability of resources. Although many countries have adopted one or more of these WHO-recommended strategies in their national cancer control plans [3–5], implementation of the new strategies has faced significant barriers, slowing the translation of policy to real-world practice [3, 6]. Unless these implementation challenges are adequately addressed, cervical cancer elimination goals will not be met, resulting in millions of preventable deaths.

Once a screening strategy has been adopted into a national screening program, a major barrier to successful implementation, scale-up and sustainment of cervical cancer prevention programs has been insufficient guidance on best practices for resource and operations planning. “Resource and operations planning” is the set of decisions required to implement a screening approach, such as determining the amount and allocation of staff and equipment and how and when to transport samples and results. Uninformed choices in resourcing and operations risk causing early failures in implementation, leading to reduced enthusiasm for new programs and even reversion to previous ineffective program plans. While these choices are often treated informally, we can facilitate data-driven resource planning and decision-making by “virtual experimentation” to examine the alternative implementation strategies using discrete event simulation (DES).

DES models have been used extensively in industrial engineering and in healthcare to improve operational efficiency and effectiveness by simulating "what-if" scenarios before investing in process changes [7–16], including in screening applications [8, 11] and in other applications in LMIC countries [17, 18]. DES models can represent health service systems by modeling patients, samples, and other entities as they move through a process [19]. A process consists of a series of steps such as registration, sample collection, sample testing, and results delivery. The representation is sufficiently detailed to simulate interactions among patients/samples through resource-constrained process steps. For example, by simulating all the patients and samples as they step through the process, a DES model can measure the queues that result when patients wait to see a busy doctor and compute how long samples wait to be processed by a laboratory. By modeling the complex dynamics that lead to these bottlenecks and unintended consequences, DES models can explore the relative impact of both high-cost solutions such as building new physical space or hiring new staff against lower cost solutions such as workflow redesign and patient/staff scheduling. These examples are analogous to decisions facing LMIC stakeholders weighing the desirability of increasing coverage of cervical cancer screening and follow-up against the feasibility of meeting the new demand without a significant increase in resources.

The overarching goal of this project is to demonstrate the value of DES in implementation research and practice, particularly to support operational planning for sustainable and resilient delivery of healthcare interventions. While we apply DES to guide decision-making and resource utilization for cervical cancer prevention programs, these multi-level planning and implementation challenges represent common barriers to the successful scale-up of many preventative health interventions worldwide. DES represents a broadly applicable tool to address complex implementation challenges across settings and health interventions—how to make effective and efficient operational and resourcing decisions to support program adaptation (i.e., make more suitable) to local constraints in order to foster fidelity and resiliency.

## Methods

### The Peruvian context and data

In Iquitos, a city of ~ 400,000 in the northern Amazon basin region of Peru, we conducted a participatory action research study to develop, implement, and test stakeholder-driven strategies for cervical cancer screening and treatment [20]. As part of this Proyecto Precancer Study, a difficult barrier emerged: making effective and efficient operational and resourcing decisions so that the new screening program is better adapted to the local constraints and demand. Available tools from the WHO [21, 22] were essential in identifying “what” to do but provided insufficient guidance for “how” to do it. To support such decisions, we decided to build a DES model of the screening process to carry out virtual experiments to assess the screening system’s performance at various resource levels.

**Fig. 1:**
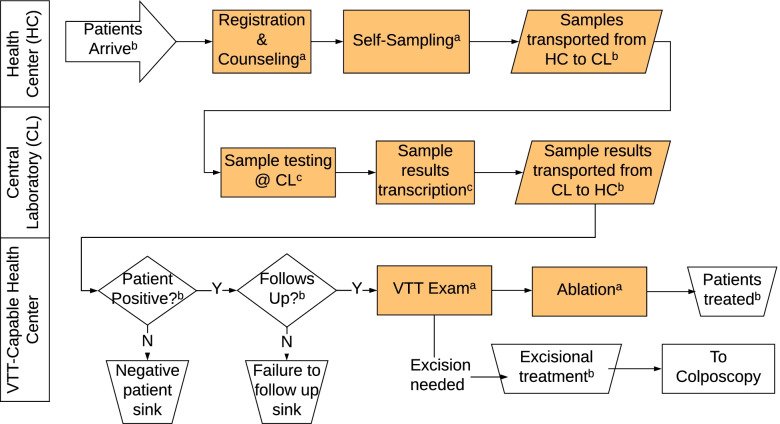
Process for HPV screen and treat, as represented in the DES model. Data estimated from **a** time and motion study, **b** electronic screening registry, and **c** direct observation

As part of the Proyecto Precancer Study, we carried out a process mapping exercise [23, 24] whereby multi-level stakeholders, including obstetras, doctors, colposcopists, administrators, and health authorities from the regional Ministry of Health, visually outlined and expanded upon the major steps and processes in the newly developed HPV-based screen and treat program for cervical cancer prevention. The process map (Fig. 1) was then used to build a simulation model, which was parameterized with real-life data from a time and motion study and screening registry.

As part of a time and motion study, we collected informed consent from patients and providers to directly observe and record time spent on different in-clinic activities and procedures at the major steps along the HPV-based screen and treat program process; data were collected at three clinics over 14 days (Appendix). For screening, we recorded the following times (in minutes): patient arrival, pre-screening registration and counseling, and HPV sample collection (clinician or self-collection). For the follow-up visit, we recorded the following times: arrival; pre-treatment registration, counseling, and consent; visual examination to determine thermal ablation (TA) eligibility; TA procedure; post-treatment counseling; and disinfection of TA probes. For HPV laboratory testing, we used direct observation to measure time (in minutes) for sample accessioning, processing, testing, and recording of results. We used our electronic screening registry to estimate the turnaround time (in days) from sample collection to HPV test completion in the central laboratory and from HPV result availability to conduct of visual triage to treatment (VTT) examination (for HPV positive women). Positivity rates, patient arrival rates, follow-up losses, and excision requirement rates were also estimated from the electronic screening registry.

### The discrete event simulation model

A DES model was created to represent the process maps generated by multi-level stakeholders in Iquitos (Fig. 1). The movement of patients and samples is simulated as they move through the process steps. Each step (shown in orange in Fig. 1) takes some amount of time to complete (a stochastic value drawn from a distribution) and has a fixed capacity (such as two staff who can process parallel registrations or an instrument with four testing wells). When a patient or sample finishes one step, it waits until the next step is available, potentially generating a queue if the next step remains busy. When the process branches—such as when a test may be positive or negative—the branch is chosen by drawing from a probability distribution. Thus, running the model simulates realistic variations in patient arrivals, queues, activity durations, and outcomes, accounting for the resource constraints in all parts of the modeled system.

The parameters for the model are designed to represent the process implemented in Iquitos. Processing time distributions are estimated from the time and motion study and the screening registry by fitting a distribution to the data (Appendix). In all cases, either a triangular or PERT distribution (based on the beta distribution and defined by the minimum, most likely, and maximum values of a variable) [25] was chosen because it provided a good fit based on the Kolmogorov–Smirnov test and is easily interpretable. The inputs and assumptions for the simulation model are described in Table 1.

**Table 1:** Baseline scenario assumptions

Parameter	Assumption
Central lab testing capacity	Two 4-well GeneXpert machines can process up to eight samples at one time.
Central lab testing time (for sample analysis and results processing)	Triangular distribution in which the most likely value (central parameter) is 65 min to complete the testing process and output the results; 5 min for results processing (see the Appendix section for all the parameters of the distribution).
Scheduled hours of operation for CxCa screening	3 h per day, 5 days per week.
Demand for screening	4000 women per year, with Poisson-distributed arrival times.
Batching for transport	Samples are collected, stored, and sent from a health center to the central lab once a week on Wednesdays; results are returned on the same schedule.
Additional processing times	See the Appendix section for additional processing time parameters.

The model was verified to ensure that it accurately represented the processes mapped by stakeholders, through detailed examination and by testing for reasonable outputs in a range of standard and extreme input parameters. Since the process has not yet been fully implemented in Iquitos, we followed the same approach as previous authors [16, 26]: the model was validated through stakeholder consultations instead of against data from Iquitos. Specifically, stakeholders were consulted through workshops in which they participated in the initial process mapping and later reviewed, refined, and approved the final process maps. Stakeholders were also shown the modeling results in a presentation and asked whether the results matched their experience with the processes. It is also important to note that the first part of the process, from sample collection to HPV testing, was fully implemented but with inconsistent processes and levels of demand, while the second part of the process was in a pilot phase. The model was implemented in SIMIO version 11, an industry standard software package for this type of simulation.

### Simulating health system performance under various scenarios

The model enables “virtual experiments” that evaluate the screening system’s performance under hypothetical “what-if” scenarios. Each scenario represents a potential change in the screening system’s resources, such as more staff or fewer operating hours. These virtual experiments allow decision-makers to evaluate the screening system's adequacy to meet planned targets and its resilience to change and to test potential resourcing approaches, providing a basis for informed, data-driven implementation plans. Specifically, the performance of the health system is estimated in four experiments to investigate: (1) how many women can be screened with the baseline level of resources, (2) the resilience of the screening system to changes that make process steps take longer or take less predictable amounts of time, (3) the resources that would be required to scale up to meet a higher demand for screening, and (4) the resilience of the screening system to larger disruptions such as unavailable equipment or personnel.

The baseline scenario represents the current status of the screening system. In virtual experiments, the assumptions are varied from the baseline to represent a set of “what-if” scenarios, such as higher demand or slower processing times. The baseline scenario assumptions are provided in Table 1. Each experiment includes a series of “what-if” scenarios, which each change one or more of the baseline assumptions, as shown in Table 2. For example, to investigate the consequences of higher demand, one parameter (demand) is varied to create three “what-if” scenarios, which increase the demand from the baseline of 4000 to 4700, 5500, and 6000 women per year.

### Outcome measures

To compare scenarios, several measures of performance, or key performance indicators (KPIs), are defined based on the goals and constraints of the screening system. These measures are as follows:

1. Number of women screened per year
2. Maximum time between sample collection and sample testing
3. Percent utilization of key resources

**Table 2:** Detail of the "what-if" scenarios

Experiment	Description	Parameters varied	Values in "what-if" scenarios (* indicates baseline value)
Screening system capacity with baseline resources (Figs. 2 and 3)	Assess capacity to meet the increasing demand for screening	Demand for screening	4000*, 4700, 5500, 6000 women per year
Resilience to changes in processing times (Fig. 4)	Assess resilience to longer or more variable processing times	Most likely central lab testing time (central parameter of triangular distribution)	Multiplied by a factor of 1*, 1.5, 2, and 3
		Maximum central lab testing time (last parameter of triangular distribution)	Multiplied by a factor of 1*, 1.5, 2, and 3
Scaling up to meet a higher demand (Fig. 5)	Identify the resources required to meet a higher demand of 9500 women per year	Demand for screening	9500
		Scheduled hours of operation for CxCa screening per day (assumes 5 days per week)	3*, 4, 5, and 6
		Central lab testing capacity (number of GeneXpert testing wells)	8* and 12
Resilience to disruptions (Fig. 6)	Assess resilience to disruptions which result in cuts in laboratory operating hours or testing machine availability	Scheduled hours of operation for CxCa screening per day (assumes 5 days per week)	3*, 2, and 1
		Central lab testing capacity (number of GeneXpert testing wells)	4, 8*, and 12

The first performance measure evaluates how well the screening system meets its goal to screen and treat the target population. It is computed by measuring the number of women whose results are reported, in the full year of simulated operations. The second measure tests whether the screening system meets a key constraint: the maximum time a sample spends in the system must not be greater than 14 days, or it expires and cannot be used (according to the HPV test manufacturer). It is computed by measuring the amount of time every sample spends from collection until testing, then finding the maximum across all the simulated samples. The third measure helps to identify parts of the process that are under-resourced or over-resourced by measuring how frequently a resource is being utilized, as a percentage of its scheduled time available. Specifically, percent utilization is defined as the percentage of a resource’s scheduled available time that is actively spent doing a process. For example, if a resource is scheduled to be available for 8 h a day and the resource is actively used for 4 h, then percent utilization would be 50%. When a process step’s utilization approaches 100%, it becomes a bottleneck and limits flow through the system. Identifying bottlenecks reveals the capacity limit of the screening system and shows where more resources are needed if capacity is to be expanded or resilience is to be enhanced.

The KPIs are computed by the model over one full year of screening operations, which is sufficient to capture all relevant variability and seasonality. This 1-year period was determined by comparing the performance of the model for 2 and 3 years, which produced equivalent results. Because the model parameters (such as processing times) are drawn from a distribution, the KPIs also vary for each simulation run. Therefore, each “what-if” scenario is simulated 100 times, and the results are reported as a box plot in which the median line, interquartile range box, range line, and outlier marks are shown. This number of repetitions was determined after a comparison of the results with those from 300 and 500 repetitions.

## Results

The following sections describe each of the four virtual experiments, described above and summarized in Table 2.

### Screening system capacity with baseline resources

**Fig. 2:**
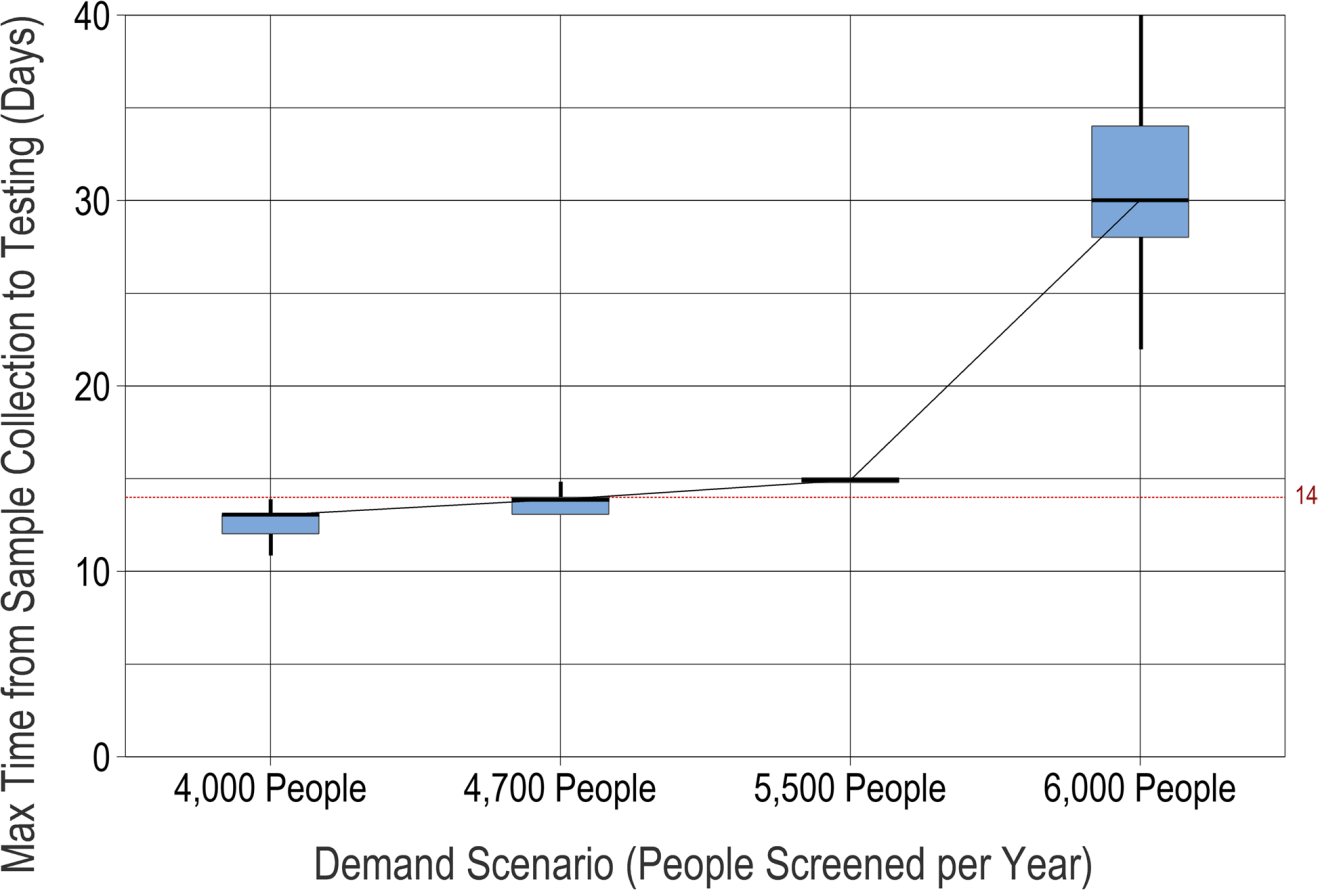
Maximum time from sample collection to testing, with baseline resource levels and increasing demand. For each "what-if" scenario (point on the *x*-axis), results from 100 simulation runs are reported on a box plot showing the median (horizontal line), interquartile range (box), range (whiskers), and outliers (asterisks)

A fundamental question is whether the screening system has the capacity to meet the target demand for screening. In addition, this target could change—if, for example, the population increases, the coverage area expands, or more women choose to be screened in one year. Therefore, various levels of screening demand were simulated to determine the capacity of the screening system with its baseline level of resources. Figure 2 shows the maximum time that any sample spends in the system for scenarios with different yearly demand levels. Recall that the samples must spend less than 14 days between collection and testing. Figure 2 shows that the screening system is able to reliably meet the baseline demand (4000 women per year) without samples waiting more than 14 days, but for higher demand, the wait times increase slowly and then rapidly when demand rises to 6000 per year. Based on these results, the screening system’s maximum capacity is around 4700 women per year. This leaves little room for expansion of coverage with current resources.

The models also enable us to examine the “bottlenecks” in the screening system—the process steps whose capacity limits the overall capacity of the system. The stakeholders believe, and our analysis confirms, that the central laboratory is the main bottleneck, since all samples from all 17 health centers must be processed there. The central laboratory has two key resources: the GeneXpert machine that runs the test on the samples and the technician who transcribes the GeneXpert output into a result that can be easily interpreted by workers and patients at the health centers. Analyzing how “busy” these resources are thus provides insight into the screening system’s performance as demand increases.

**Fig. 3:**
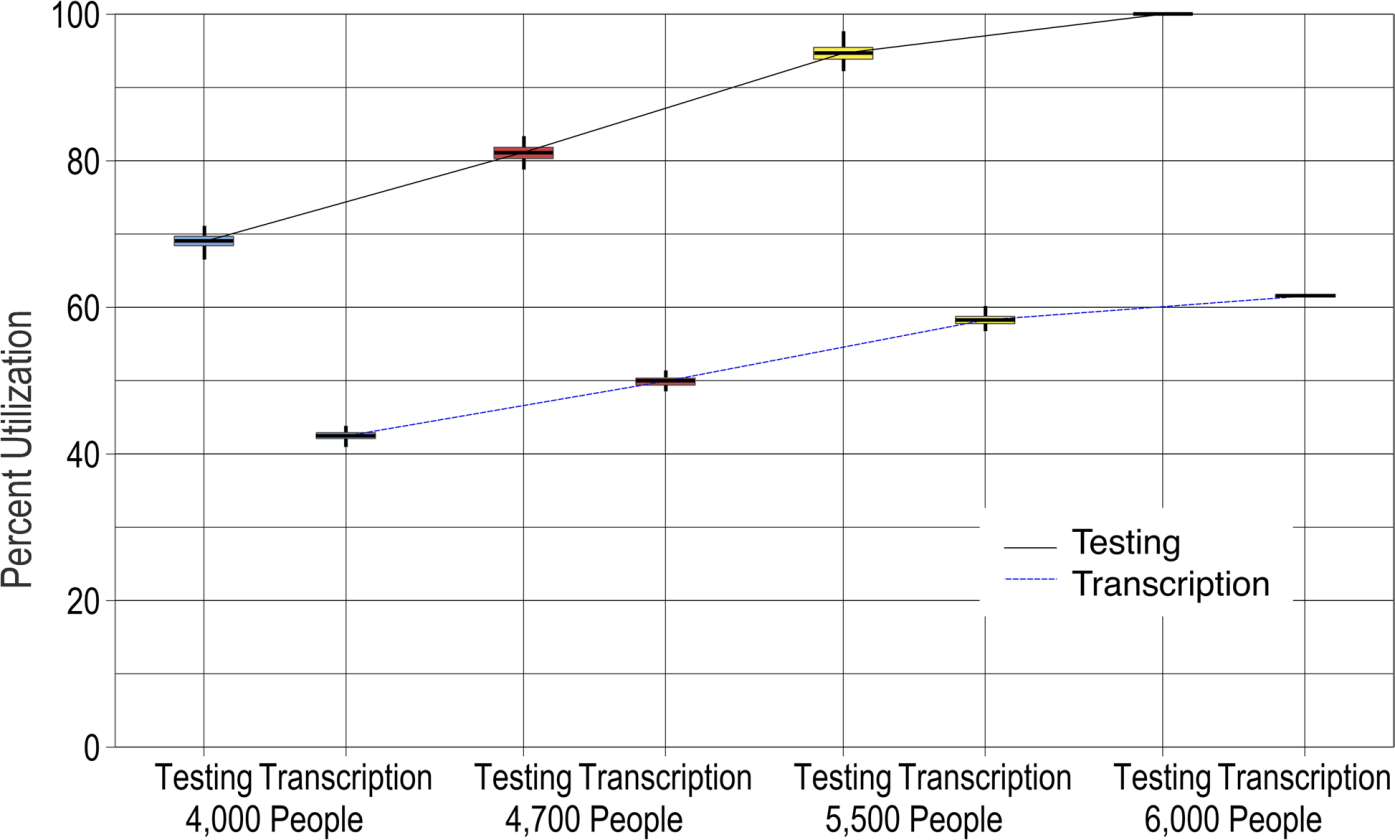
Percent utilization of two central lab resources, the GeneXpert and the laboratory technician’s time transcribing its results, with baseline resource levels and increasing demand. For each "what-if" scenario (point on the *x*-axis), results from 100 simulation runs are reported on a box plot showing the median (horizontal line), interquartile range (box), range (whiskers), and outliers (asterisks)

Figure 3 shows the percent utilization for each of these central laboratory resources. Percent utilization refers to the percentage of time that the machine is running (or busy) during the time that the machine is scheduled to run. (For example, if a machine runs 3 h per day and sits idle for 3 h a day during a 6 h scheduled availability time, then the percent utilization rate would be 50%.) For the GeneXpert machine, utilization grows from a comfortable 70% for the target demand to an unmanageable 100% for the highest demand of 6000 women per year. This helps to explain the previously observed large growth in the time a sample spends waiting: the GeneXpert machine is always busy, causing samples to pile up waiting while the machine tries to work through a growing backlog. This high utilization indicates that the GeneXpert is acting as a bottleneck, limiting the capacity of the overall screening system.

### Resilience to changes in processing times

Processing times were measured based on the current screening system operations, but they might change if, for example, new employees perform them or a process is rolled out to new locations. Therefore, the screening system performance is analyzed for processing times that are *longer* (average processing time increases) and for processing times that are more variable (*wider* distribution around the mean). Because the central laboratory is the main bottleneck, we changed the processing times for the result transcription step, which is performed by a laboratory technician after the sample is analyzed in the GeneXpert. (The GeneXpert machine processing time is not heavily influenced by human behavior and is thus less susceptible to human error, so it was left constant.) The baseline demand of 4000 people is used throughout these scenarios.

**Fig. 4:**
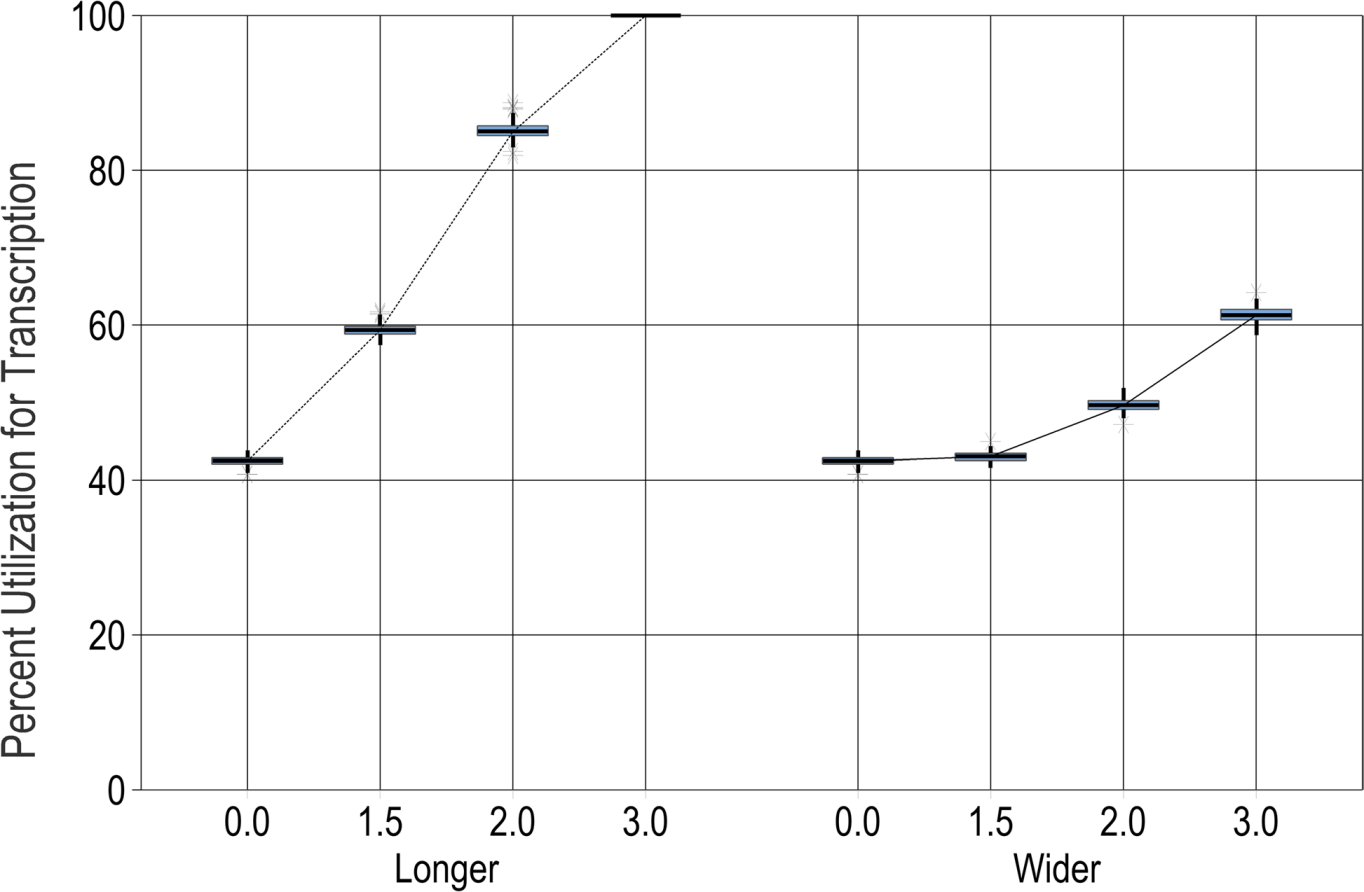
Percent utilization of the bottleneck resource, the laboratory technician’s time transcribing results, as processing times grow longer (left side) or more variable (wider; right side). For each "what-if" scenario (point on the *x*-axis), results from 100 simulation runs are reported on a box plot showing the median (horizontal line), interquartile range (box), range (whiskers), and outliers (asterisks)

The left side of Fig. 4 shows that, as expected, longer processing times lead to increasing utilization for the results transcription step. The right side of Fig. 4 shows similar but slower increases when processing times become more variable, because in this case, some samples will take much longer to process but some will also take much less time. This analysis can provide a tolerance limit on processing times for key steps. If result transcription takes twice as long, then the screening system performance is still tolerable, but if it takes three times longer than expected, a rapid performance degradation occurs because the scheduled utilization of a bottleneck process reaches 100%.

### Scaling up to meet a higher demand

The models can also provide decision-makers with information on how to scale up a system’s capacity to meet a higher demand. The scaled-up demand scenario analyzed here is 9500 people per year, more than double that of the baseline demand scenario (4000). Since the central laboratory is the rate-limiting step for this screening system, and analysis has already shown its capacity to be about 4700, meeting a higher demand requires increasing the number of lab resources, such as operating hours or testing wells. Specifically, the following scenarios explore increasing the number of hours in which the lab is scheduled to process cervical cancer samples, and/or increasing the number of testing wells from 8 to 12 (corresponding to one additional GeneXpert machine).

**Fig. 5:**
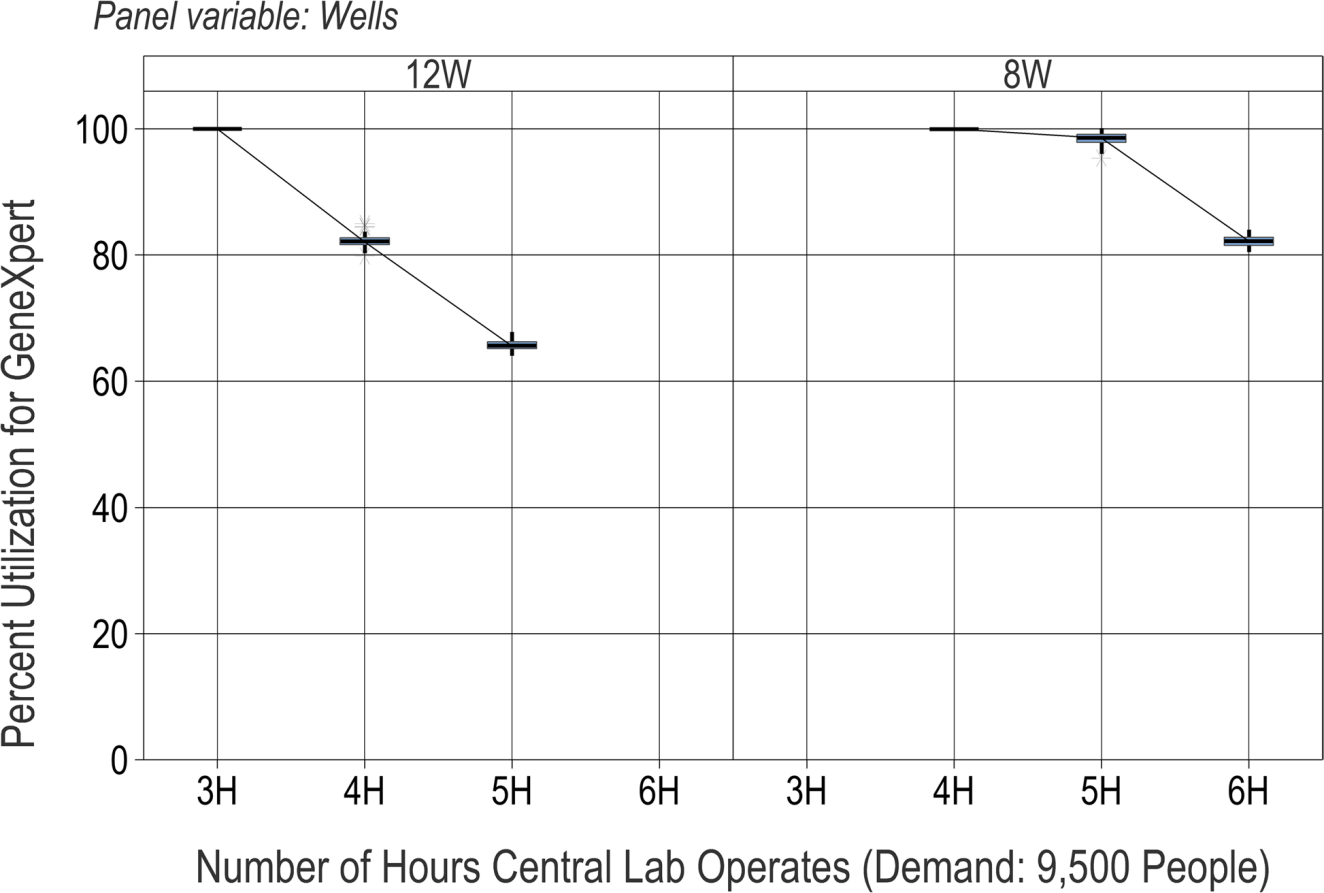
Percent utilization for various testing (GeneXpert) resource levels (operating hours, number of wells) with scaled-up demand. For each "what-if" scenario (point on the *x*-axis), results from 100 simulation runs are reported on a box plot showing the median (horizontal line), interquartile range (box), range (whiskers), and outliers (asterisks)

Figure 5 shows the percent utilization of the bottleneck resource, the GeneXpert. With the current GeneXpert capacity of 8 testing wells, adding 1 or even 2 h of lab time (increases of 33% or 66% of capacity) is insufficient to meet the higher demand, since the percent utilization remains near 100% and the sample time from collection to processing remains above 14 days. The lab time must be doubled (to 6 h) to meet the higher demand. If a GeneXpert machine is added (for a total of 12 testing wells), an increase of only 1 h of lab time is required to meet the demand. Thus, if decision-makers want to more than double the target screening capacity, they have multiple options: they may significantly increase operating hours, or they may purchase additional GeneXpert machines and modestly increase operating hours. A third alternative is to implement a batched testing platform with a higher capacity; in future work, a modified DES model could identify the threshold at which the increased demand makes batch processing efficient rather than wasteful.

### Resilience to disruptions

**Fig. 6:**
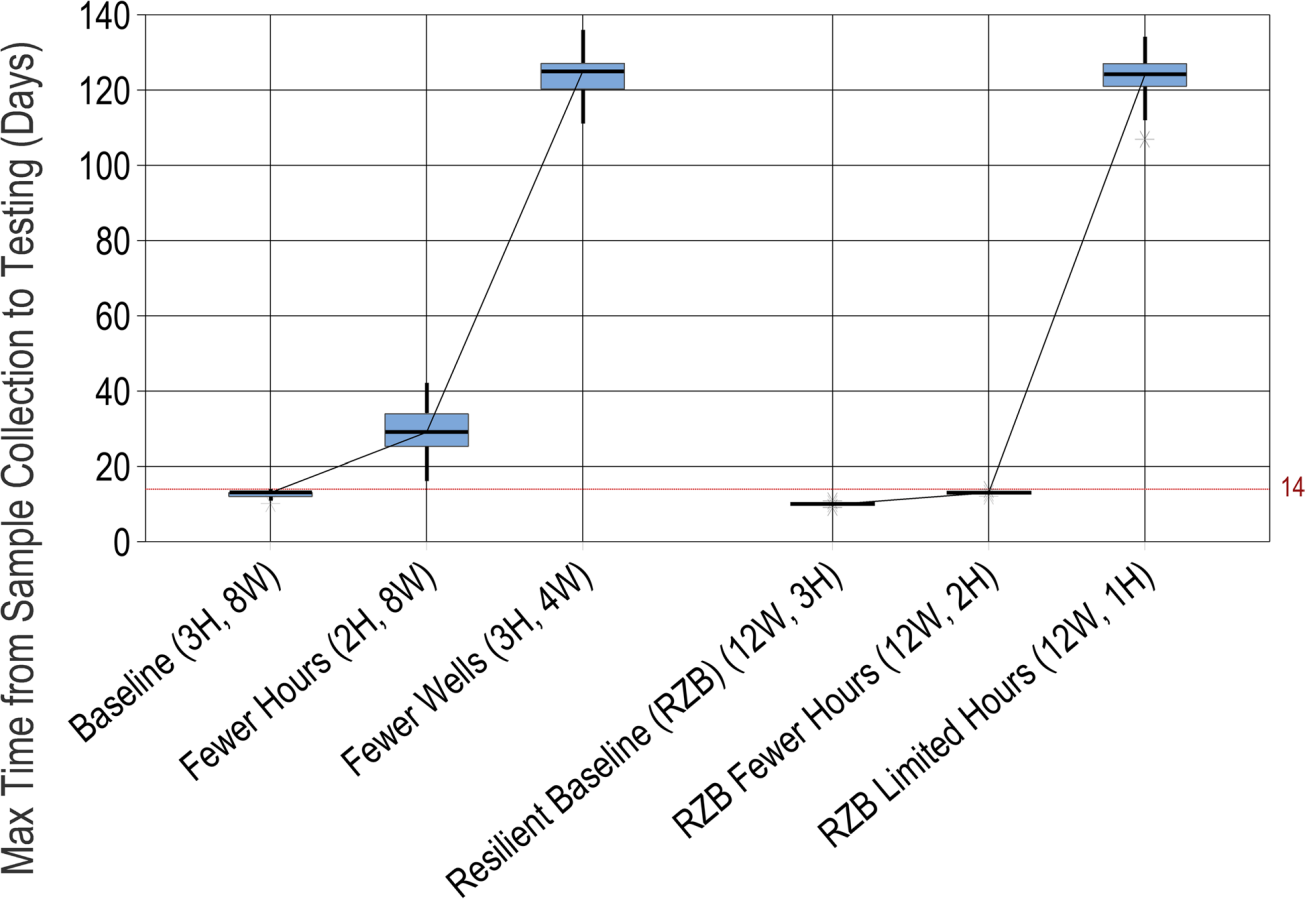
Maximum time from sample collection to testing, for various disruption scenarios. For each "what-if" scenario (point on the *x*-axis), results from 100 simulation runs are reported on a box plot showing the median (horizontal line), interquartile range (box), range (whiskers), and outliers (asterisks)

Models can also enable decision-makers to quickly understand the potential impact of disruptions on health system performance. For example, GeneXpert machines or lab time may need to be diverted away from cervical cancer screenings toward a more immediate public health need in the case of an outbreak, such as COVID-19 or TB. In such cases, the availability of the lab technicians and/or the GeneXpert machines for cervical cancer screening might decrease. Figure 6 shows the impact of cuts in both hours and GeneXpert wells on the time from sample collection to testing (which must not rise above 14 days). The results show that any reduction in GeneXpert machine availability or in the laboratory’s available hours (e.g., due to human resource constraints or diverted resources) would result in unacceptable performance, such that screening targets could not be met. Adding an additional GeneXpert machine would provide some resilience to disruptions, enabling adequate performance with hours cut by 33% (but no more). This small selection of scenarios demonstrates how these models can enable decision-makers to explore the impact of various types of disruptions, so that goals can be adjusted accordingly, or resilience can be built into the health system.

## Discussion

This paper aimed to demonstrate the value of DES in the implementation science research and practice. In this context, data were collected to estimate, for example, sample processing times, but human-involved processes are always susceptible to unexpected changes. Modeling longer and more variable processing times showed worse health system performance and enabled an understanding of the system’s processing time tolerance limits. Health systems are also susceptible to larger disruptions such as curtailed resources (e.g., such as experienced during the COVID-19 pandemic) or increased demand (e.g., such as regional scale-up or screening campaigns). Our results showed that this screening system lacks resilience to both scenarios and identified the resources required to improve performance in such cases. These are valuable but not obvious insights. Our example of HPV-based cervical cancer screening is specific to this setting in Iquitos, Peru, but similar models can be developed for other settings or healthcare delivery interventions. Discrete event simulation provides decision-makers with a powerful tool to work through many potential scenarios and constraints. Decisions on the levels of resources needed to meet various levels of demand are often too complex to analyze with “back of the envelope” calculations. Similarly, even if decision-makers have a firm understanding of their baseline scenario, they may be unable to accurately predict the effect of a resource disruption or a surge in demand or may even misestimate the severity or timing of such effects. For example, we found that increasing the screening demand by about one quarter could easily be accommodated, but further increases overtaxed the screening system and led to poor performance. Identifying the threshold at which a health system becomes overwhelmed, and the resources required to mitigate this problem is not intuitive, especially in multilevel and interconnected health systems. This paper has demonstrated a few of the ways in which discrete event simulation can mitigate some of these challenges.

For the case of HPV-based cervical cancer screening in Peru, our results show that the current pilot-tested screening system in one micro-network can meet its target capacity. That is, under the current baseline conditions, the screening system has enough capacity to reach the annual target screening goal while maintaining a 14 day results turnaround. However, efforts are now ongoing to scale it up to additional micro-networks, as well as new regions of Peru, which would place a greatly increased demand on the laboratory and potentially on other parts of the screening system. Our results show that additional resources must be devoted to avoid major service disruptions: additional laboratory hours devoted to HPV testing and/or additional testing instruments. Other generalizable real-world scenarios that represent threats to meeting the program goals, such as curtailed/diverted resources or highly variable screening services, showed similar results. Importantly, however, the model also highlights the effect of different tradeoffs such as adding more machines compared to more technician time, thus helping to identify efficient solutions to maintain resilience and sustainability in the dynamic screening system. Moreover, the models can be used to identify any new bottlenecks that may arise, such as the availability of precancer treatment providers, as the screening system resources are changed.

The insights from this specific case example can be extended in several ways. First, models can be tailored and parameterized to represent other health systems (such as HPV testing in a different region or another type of health service delivery process in the same region). Such a model would support specific decisions on resource levels required to meet changes in the health system, enhance resilience, and/or scale up services to meet increased demand or a broader coverage goal. Second, such specific models could be generalized to represent a broader range of situations (such as HPV testing in any LMIC setting). Such a model would support the development of broader guidelines that identify important tradeoffs, bound resource requirements, and guide the development of implementation plans. Third, the development and validation of the models facilitate stakeholder engagement and shared decision-making across implementation scientists and practitioners to capitalize on their respective expertise in translating interventions sustainably into real-world practice.

The use of discrete event simulations is particularly valuable when incorporating a new process into a health system, such as a new technology like COVID-19 testing [27]. For example, while there is clear policy-level guidance on the decision to adopt HPV testing technology [21, 22], much less guidance is available on the operational planning aspects that are critical to effectively and efficiently meeting this demand. As described above, tools such as discrete event simulation models can provide general “rules” or boundaries to inform decision-making around the operational aspects of healthcare service delivery. For example, our “what-if” scenarios demonstrate the importance of balancing resource efficiency against the ability to maintain service resilience to different kinds of disruptions. More specifically, our case demonstrates how the models can support evidence-based decisions on how to plan resources for resilience and/or scale-up.

There are several limitations to this study. First, data collection was based on early experiences from one micro-network; thus, continued work in this area will be informative when additional clinics and laboratories can be included, and when similar models can be developed for related settings. While the overall steps in HPV-based screening are similar across most LMIC settings, there are many implementation options to consider, such as batch-testing and point-of-care testing, which were not modeled here, and remain part of our future work. Second, the DES models and “what-if” scenarios required assumptions about how processes operate or *would* operate if they were implemented, some of which could not be validated against empirical data. Nevertheless, our results demonstrate how these models lead to useful insights, because they are not intended to be predictive but rather to gauge the relative impact of different process choices on system performance. Data and assumptions from our single empirical setting have already identified key tradeoffs, and future work in different settings could enable the development of more generalizable principles that could be applied more broadly across diverse settings.

## Conclusions

Discrete event simulation models thus fill an essential niche in our arsenal of modeling approaches. Decision analytic modeling has proven critical for getting new technologies into national health plans [28, 29]. However, once a decision to adopt has been made, the operational implementation decisions can be studied in a similar way to facilitate scale-up and sustainability. Our discrete event simulation models focus on planning the processes and operations critical to adequate delivery of services with the new technology. This aspect has received less attention but can fill a critical gap in moving evidence-based interventions into routine practice beyond an implementation study, especially in LMICs that often struggle with constant changes in human capacity, supply chain disruptions, and other resource shortages. As our community works toward aspirational goals such as cervical cancer elimination, transitioning from the many pilot and demonstration style projects to regional and national scale-up, synthesizing the operational data from these studies in a more general DES model of screening service delivery would enable broad dissemination and use of the collective global experience to accelerate the scale-up of sustainable programs.

## Data Availability

As study data is finalized, and after publication, data inquiries can be submitted via the “contact” section of the Proyecto Precancer study website https://proyectoprecancer.com/.

## Appendix

### Details of the time and motion study and HPV laboratory testing

The time and motion study consented providers and patients to directly observe and document the time spent during each activity. We observed HPV screening registration and sample collection in 3 primary health centers for 3 days each, with a total of 35 screening encounters. The time from HPV sampling to result was estimated via date stamps on the registration forms and the HPV results forms (and confirmed with time stamps on the GeneXpert instrument). For the visual triage to treatment (VTT) follow-up, only 2 primary health centers were providing this service. The time spent on pre-defined activities was recorded on standardized forms at both centers over 10 days for a total of 71 observed examinations. Table 3 details the procedure and activity times for process steps estimated from time and motion studies.

**Table 3:** Procedure and activity times (in minutes) for process steps estimated from time and motion studies. Note that the “ablation” process time is the sum of the ablation procedure and post-counseling durations, and the “VTT” process time is the sum of the VTT procedure and registration/counseling durations

Step	Distribution	Minimum	Most likely	Maximum
Registration and counseling	PERT	0	12.159	15.309
Self-sampling	Triangular	0	1	4.297
Ablation procedure	PERT	0	0.258	9.144
Ablation post-counseling	PERT	0	0.452	24.065
VTT procedure	PERT	0	0	96.648
VTT registration and counseling	PERT	0	3.239	18.172

For HPV testing, samples were collected in 1 mL of standard transport medium (Digene), as liquid-based cytology medium (e.g., ThinPrep or SurePath) was not available and within the budget for the project. Samples were registered as being delivered from the health centers to the laboratory in a notebook, and two carbon copies of the triplicate registration form were left in the laboratory. The original copy was returned to the primary health center. Once the samples arrived in the laboratory, a 500-μL aliquot was removed using the transfer pipette included in the GeneXpert kit and placed in 5 mL of phosphate-buffered saline according to Mbulawa et al. [30]. After a brief vortex, 1 mL of the PBS-diluted sample was added directly to the GeneXpert cartridge. Samples were stored at room temperature. The manufacturer’s instructions indicate to test within 2 weeks of collection, and our data suggests that > 95% met that criterion (median 7 days, IQR 4–13 days).

## Abbreviations

WHO: World Health Organization
LMIC: Low- and middle-income countries
DES: Discrete event simulation
HPV: Human papillomavirus
TA: Thermal ablation
KPI: Key performance indicators
VTT: Visual triage to treatment
